# Supplementary material for: Familiar face + novel face = familiar face? Representational bias in the perception of morphed faces in chimpanzees
Source: PeerJ. 2016 Aug 4;4:e2304. doi: 10.7717/peerj.2304 (PMC4991860; doi:10.7717/peerj.2304)
Supplement: Supplemental Information 1 [file peerj-04-2304-s001.pdf]

Supplementary information

Title:

Familiar face + novel face = familiar face? Morphed face recognition in chimpanzees

Yoshi-Taka Matsuda*, Masako Myowa-Yamakoshi, and Satoshi Hirata*

*To whom correspondence should be addressed.

E-mail: matsuda@brain.riken.jp, hirata.satoshi.8z@kyoto-u.ac.jp

This file includes:

1. **Supplementary Materials**

2. **Eye-tracking data (for Figures 1 and 2 in the main text)**

3. **Cumulative histogram of fixation duration (Figure S1)**

1. Supplementary Materials and Methods

Subjects

Table S1. Relative dominance (chimpanzee # is dominant/subordinate/equal over familiar face #; M and F denote male and female, respectively)

	familiar face 1 (M)	familiar face 2 (M)	familiar face 3 (F)
chimpanzee1 (F)	subordinate	subordinate	(mother)
chimpanzee 2 (F)	subordinate	subordinate	subordinate
chimpanzee 3 (M)	(self)	dominant	dominant
chimpanzee 4 (F)	subordinate	subordinate	(self)
chimpanzee 5 (F)	subordinate	subordinate	equal
chimpanzee 6 (F)	subordinate	subordinate	subordinate
chimpanzee 7 (F)	subordinate	subordinate	dominant
chimpanzee 8 (M)	subordinate	(self)	dominant

Table S2. Kinship structure (familiar face # is father/mother/self/non-kin of chimpanzee #)

	familiar face 1 (M)	familiar face 2 (M)	familiar face 3 (F)
chimpanzee 1 (F)	father	non-kin	mother
chimpanzee 2 (F)	father	non-kin	non-kin
chimpanzee 3 (M)	self	non-kin	non-kin
chimpanzee 4 (F)	non-kin	non-kin	self
chimpanzee 5 (F)	non-kin	non-kin	non-kin
chimpanzee 6 (F)	father	non-kin	non-kin
chimpanzee 7 (F)	non-kin	non-kin	non-kin
chimpanzee 8 (M)	non-kin	self	non-kin

2. Eye-tracking data

For Figure 1. Visual preference

Figure (b)

Fixation duration (%)			
	familiar face	intermediate face	novel face
chimpanzee 1	22.975	35.526	41.498
chimpanzee 2	38.845	25.450	35.705
chimpanzee 3	32.691	19.791	47.518
chimpanzee 4	40.385	22.332	37.283
chimpanzee 5	11.399	32.149	56.453
chimpanzee 6	40.334	28.532	31.134
chimpanzee 7	30.320	26.420	43.260
chimpanzee 8	28.919	31.186	39.895

Figure (c)

Fixation count (%)			
	familiar face	intermediate face	novel face
chimpanzee 1	26.087	32.609	41.304
chimpanzee 2	38.889	27.778	33.333
chimpanzee 3	38.235	17.647	44.118
chimpanzee 4	25.000	25.000	50.000
chimpanzee 5	17.857	35.714	46.429
chimpanzee 6	29.268	39.024	31.707
chimpanzee 7	28.889	28.889	42.222
chimpanzee 8	24.000	28.000	48.000

Figure (d)

Saccade length (%)			
	familiar face	intermediate face	novel face
chimpanzee 1	22.391	31.687	45.922
chimpanzee 2	40.095	19.719	40.186
chimpanzee 3	36.287	16.535	47.178
chimpanzee 4	35.547	24.453	40.001
chimpanzee 5	13.570	34.482	51.949
chimpanzee 6	31.182	36.490	32.328
chimpanzee 7	27.147	26.538	46.315
chimpanzee 8	24.356	32.030	43.615

For Figure 2. Visual preference

Figure (a)

Fixation duration (%)		
	100% novel	50% novel + 50% novel
chimpanzee 1	50.103	49.897
chimpanzee 2	48.299	51.701
chimpanzee 3	66.886	33.114
~~chimpanzee 4~~	~~0~~	~~100~~
chimpanzee 5	49.475	50.525
chimpanzee 6	67.913	32.087
chimpanzee 7	63.206	36.794
chimpanzee 8	68.917	31.083

Figure (b)

Fixation count (%)		
	100% novel	50% novel + 50% novel
chimpanzee 1	52.941	47.059
chimpanzee 2	42.857	57.143
chimpanzee 3	71.429	28.571
~~chimpanzee 4~~	~~0~~	~~100~~
chimpanzee 5	69.231	30.769
chimpanzee 6	61.111	38.889
chimpanzee 7	63.636	36.364
chimpanzee 8	33.333	66.667

Figure (c)

Saccade length (%)		
	100% novel	50% novel + 50% novel
chimpanzee 1	54.103	45.897
chimpanzee 2	57.072	42.928
chimpanzee 3	64.132	35.868
~~chimpanzee 4~~	~~0~~	~~100~~
chimpanzee 5	42.672	57.328
chimpanzee 6	38.707	61.293
chimpanzee 7	49.272	50.728
chimpanzee 8	62.116	37.884

3. Cumulative histogram of fixation duration

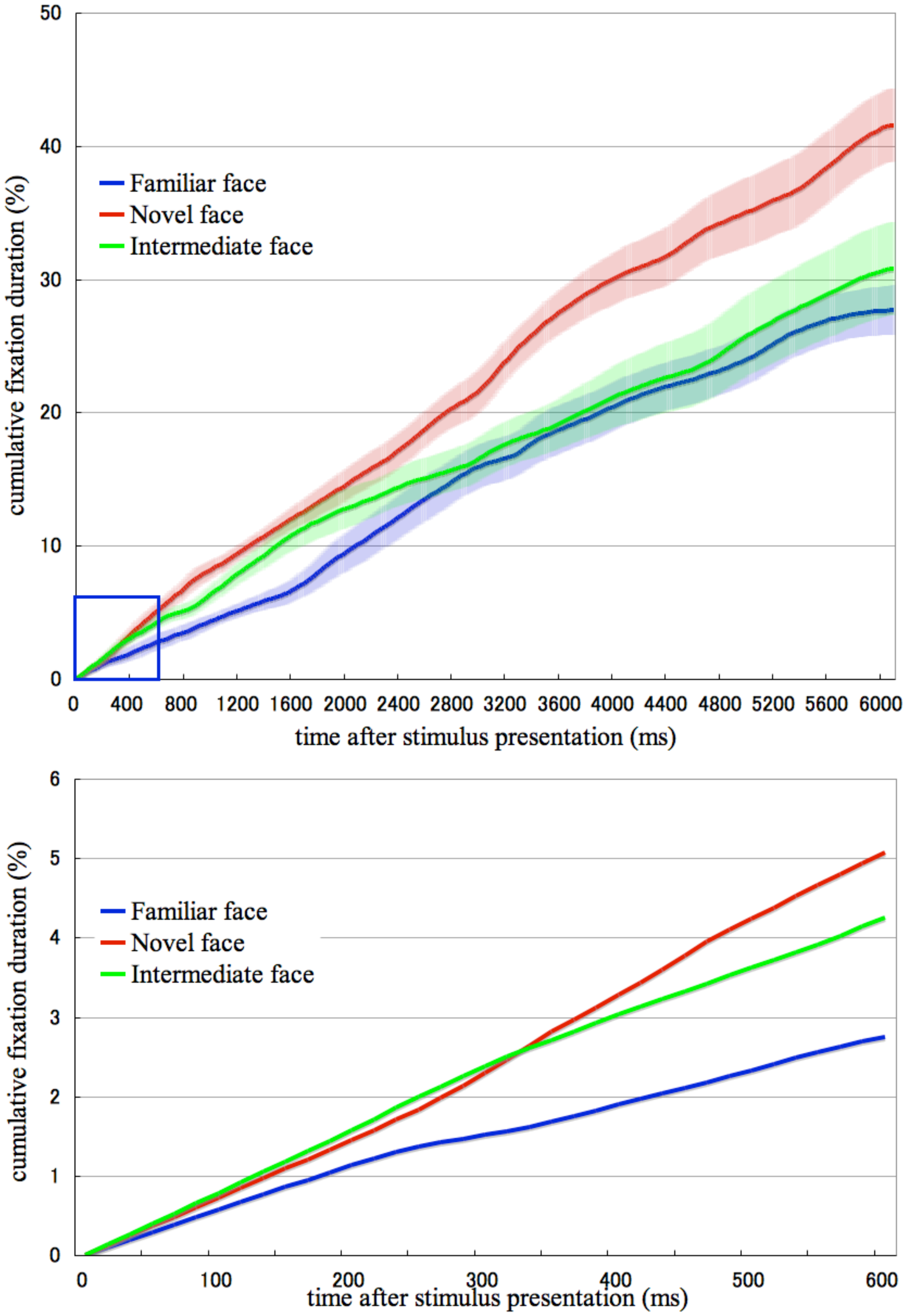

Figure S1. Cumulative histogram of fixation duration after stimulus presentation. Upper panel: The figure depicts the cumulative fixation duration ($N = 8$, average) when

looking at familiar (blue line), novel (red line) and intermediate (green line) faces after stimulus presentation. Cumulative fixation durations were normalised at the end of stimulus presentation (6,000 ms) for each subject to calculate proportions among three types of facial stimuli. Solid lines depict mean; pale zones depict SEM. Lower panel: the figure depicts higher resolution of the inset (initial 600 ms; blue rectangle) in the upper panel.
